# Henoch-Schönlein Purpura Without Proven Immunoglobin A Deposition: A Diagnostic Distinction

**DOI:** 10.7759/cureus.82312

**Published:** 2025-04-15

**Authors:** Pierce W Froberg, Angelique Ruml, Joan K Fernandez

**Affiliations:** 1 Research, Private Practice, Dallas, USA; 2 Dermatology, Baylor College of Medicine, Houston, USA

**Keywords:** direct immunofluorescence, henoch schönlein purpura, ig a vasculitis, immune complex deposition, immunoglobulin a, palpable purpura

## Abstract

Henoch-Schönlein purpura (HSP), or immunoglobulin A (IgA) vasculitis (IgAV), is a small vessel vasculitis that is most commonly seen in children; it is classically characterized by IgA deposition within the renal mesangium, resulting in a wide range of symptoms: palpable purpura, arthralgia, gastrointestinal symptoms, renal involvement, and, in severe cases, pulmonary complications or intussusception. Diagnosis relies upon clinical symptoms, histopathology, and direct immunofluorescence (DIF) testing to differentiate HSP from other vasculitides. DIF typically reveals IgA deposits; however, negative DIF findings do not rule out the diagnosis, which indicates the need for an adaptable diagnostic approach. Groups such as the European League Against Rheumatism (EULAR) and the American College of Rheumatology (ACR) categorize positive IgA as supportive data for an HSP diagnosis but not as a necessity.

We present the case of a 14-year-old male with progressive ascending palpable purpura, significant abdominal pain, and lower extremity edema. Histopathological analysis of the skin biopsy confirmed leukocytoclastic vasculitis (LCV); however, his DIF was negative for IgG, IgA, IgM, C3, and fibrinogen deposits. At the time of biopsy, his workup was significant for an isolated elevated alanine aminotransferase (ALT), but urinalysis and renal function testing were unremarkable. Despite a negative DIF, the patient’s findings were clinically consistent with HSP, and hence, a diagnosis was made. He was started on a prednisone taper and supportive care for management. After initial improvement, he experienced a flare warranting an additional course of steroids, which improved his symptoms. This report underscores the diagnostic challenges associated with HSP, particularly with negative DIF. While DIF serves as a useful tool in the classification of certain vasculitides, its sensitivity is influenced by the biopsy site, lesion age, and degradation of immune complexes. Providers should maintain a high index of suspicion and carefully consider histopathologic findings and laboratory data when diagnosing HSP. Further research is needed to refine the diagnostic applicability of DIF and its association with disease severity and systemic involvement.

## Introduction

Henoch-Schönlein purpura (HSP), also known as immunoglobulin A (IgA) vasculitis (IgAV), is a systemic vasculitis resulting from small vessel inflammation characterized by IgA deposition in the renal mesangium [1]. It is associated with a distinct clinical triad: palpable purpura affecting the dermis, arthralgia, and gastrointestinal involvement. Additional manifestations include lower extremity edema, hematuria, proteinuria, and, in extreme cases, intussusception or pulmonary complications [2]. HSP predominantly affects children aged four to eight years, with a slight male predominance [1,3,4]. Its reported incidence ranges from 10 to 20.4 cases per 100,000 children annually [2,3], while adult cases occur at one-tenth this rate [5]. Age at the time of diagnosis is an important factor in influencing disease severity and outcomes [1].

While infants typically exhibit mild renal involvement, adults face an increased risk of chronic nephritis and a prolonged disease course [6]. Clinical features vary across age groups, and atypical symptoms present at the extremes [3]. Children less than two years exhibit almost exclusively cutaneous and articular involvement, and younger children tend to experience a greater prevalence of purpura, glomerulonephritis, and end-stage kidney disease (ESKD) [2,3,7]. The etiology of HSP is not fully understood, but associations have been established with infectious bacterial triggers such as Streptococcus pyogenes and Helicobacter pylori, vaccinations, special food, certain drugs, and, in rarer cases, HIV [1,2,8,9]. Environmental factors, particularly seasonal variations, suggest a link between respiratory infections and HSP [4]. Seasonal peaks of incidence in colder months may suggest that respiratory infections play a significant role in the pathogenesis of HSP. Overall, no single pathogen has been deemed responsible for HSP onset [1,5]. 

Clinically, purpuric HSP lesions are mainly concentrated on the lower extremities, buttocks, calves, and ankles but can extend to the trunk, abdomen, pubis, or scrotum [1]. Lesions above the waist may indicate gastrointestinal involvement if associated with abdominal pain, though conflicting evidence challenges this association [7,10]. Rare manifestations include neurologic conditions such as headache, altered mental status, and seizure. Pulmonary complications can also arise, such as diffuse alveolar hemorrhage, interstitial pneumonia, and fibrosis [1]. These pulmonary symptoms pose substantial diagnostic and therapeutic challenges [6]. Full recovery is observed in the majority of cases without end-stage renal failure, pulmonary distress, or central nervous system complications. In the case presented in this report, the patient experienced favorable renal outcomes. The objective of this report is to explore the diagnostic challenges encountered in the management of a 14-year-old with HSP in the context of negative direct immunofluorescence (DIF) and to emphasize the importance of clinical correlation and histopathologic findings in confirming the diagnosis.

## Case presentation

**Figure 1:**
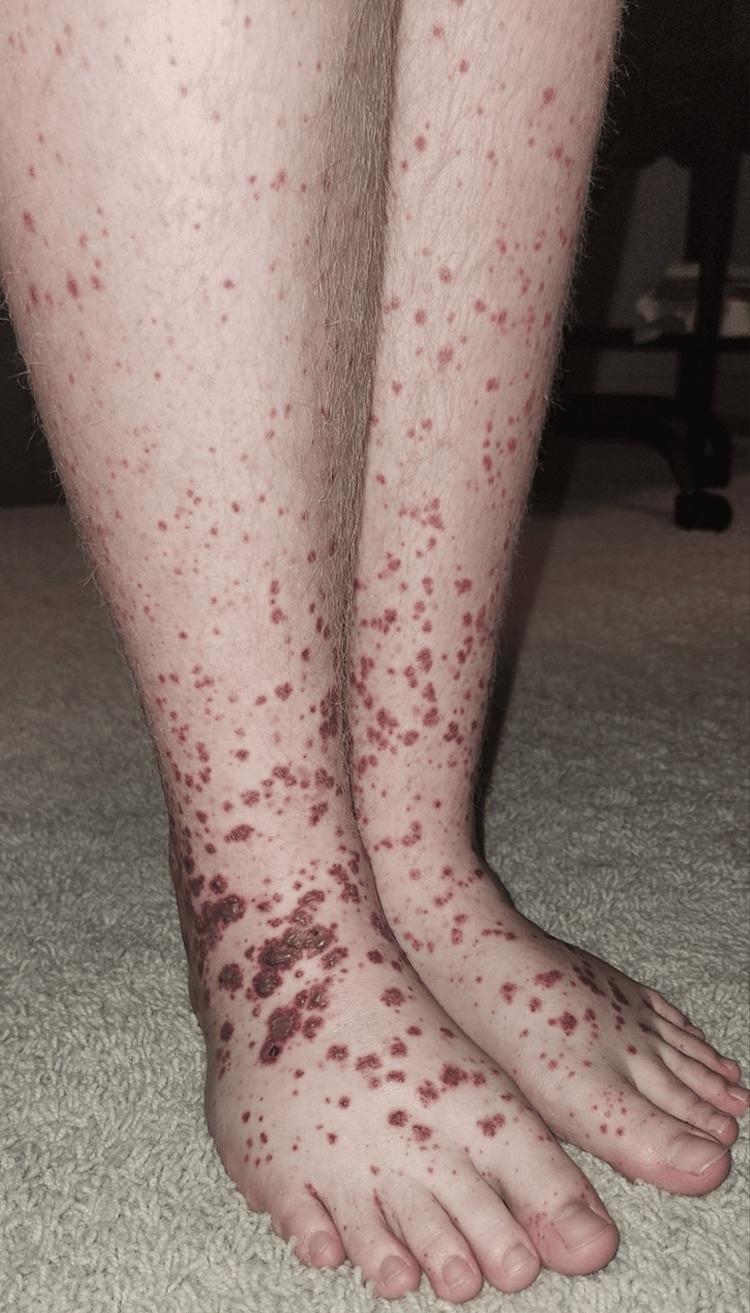
Palpable purpura of the lower extremities

**Figure 2:**
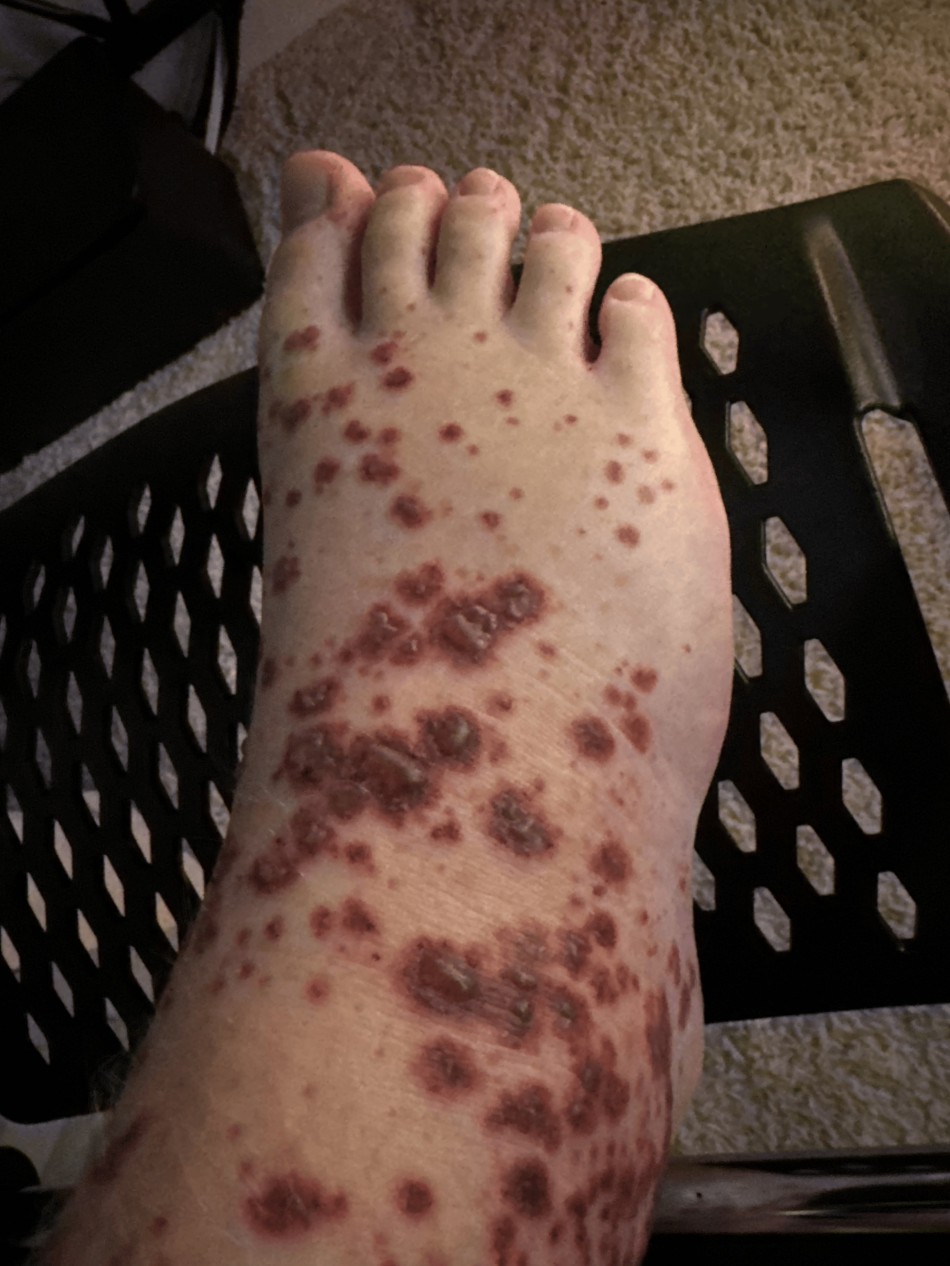
Palpable purpura of the right dorsal foot

**Figure 3:**
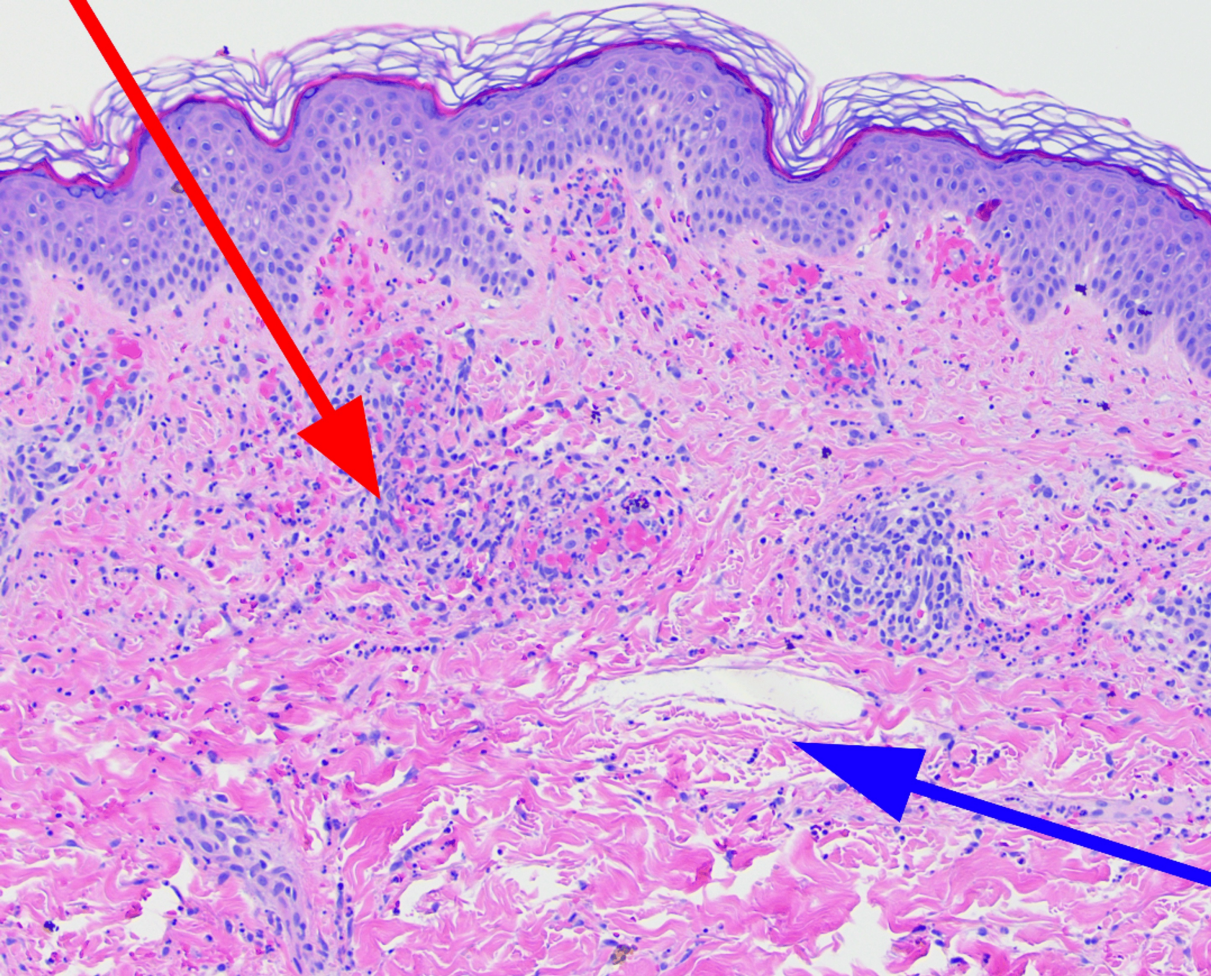
Punch biopsy showing perivascular and interstitial infiltrate comprised of neutrophils (red arrow) and extravasated erythrocytes (blue arrow)

An otherwise healthy 14-year-old male presented to Evolve Dermatology with a one-week history of a progressive rash and significant abdominal pain. The rash had initially appeared on the dorsal aspects of the feet, subsequently ascending to the medial thighs. Examination revealed palpable purpura with necrotic centers and pitting edema extending to the upper shins (Figures 1, 2). The patient's face, chest, and abdomen were otherwise clear. He maintained normal oral intake, and his guardian denied any indication of recent viral illness, recurrent systemic infection, or diabetes. At the time of initial presentation to the dermatology clinic, two 4 mm punch biopsies were obtained, including one from the right medial thigh for DIF. The differential included leukocytoclastic vasculitis (LCV) favoring HSP. Histopathology revealed LCV, while DIF was negative for the IgG, IgM, IgA, C3, and fibrinogen deposits (Figure 3).

A comprehensive metabolic panel ordered by the patient’s pediatrician one week prior had shown values within normal limits. Given symptom severity, the patient was referred to the emergency department. Laboratory findings, including a complete blood count, comprehensive metabolic panel, pulmonary workup, and urinalysis, were unremarkable. Notably, the patient’s serum creatinine and blood urea nitrogen were within normal limits, and there was no evidence of hematuria or proteinuria on urinalysis. The emergency room agreed with the diagnosis of HSP, and the patient was discharged home. Three days after the initial dermatology and emergency room visit, the patient’s guardian reported worsening swelling of the patient’s extremities and rash progression to the trunk, buttocks, and distal forearms. The patient was subsequently started on a short-term prednisone taper. At the two-week follow-up, the patient’s guardians reported improved rash and abdominal pain.

The patient completed his prednisone taper with complete resolution of his rash. However, four weeks following prednisone taper completion, he experienced another flare with tenderness to palpation, recurrent lower extremity edema, and new purpuric lesions. He denied abdominal pain, hematuria, or respiratory symptoms. A second prednisone taper was initiated. Repeat urinalysis and basic metabolic panels were obtained, revealing elevated alanine aminotransferase (ALT) (53 U/L; reference range: 7-32 U/L), while aspartate aminotransferase (AST), alkaline phosphatase, and bilirubin levels were unremarkable. Renal function was within normal limits, and urinalysis was negative for hematuria or proteinuria. Three weeks after the second follow-up, the patient returned with a recurrence of purpuric lesions but no abdominal pain. Urinalysis and a basic metabolic panel were obtained once again, with only ALT noted to be elevated (77 U/L; reference range: 7-32 U/L). No evidence of hematuria or proteinuria was detected, and a third prednisone taper was initiated. The patient continues to undergo regular monitoring for renal abnormalities and recurrent purpuric flares.

## Discussion

Since clinical presentation alone is not always sufficient for diagnosing HSP, a comprehensive evaluation incorporating laboratory findings, histopathology, and clinical context is essential. A definitive biopsy for HSP typically demonstrates LCV, characterized by perivascular neutrophilic infiltrate with karyorrhexis and evidence of vascular damage [11]. DIF for HSP commonly reveals granular deposits of IgA and immune complexes around affected and unaffected vessels [12]. While evidence suggests a strong correlation between IgA deposits and HSP, some HSP cases with classic presentation lack IgA deposition on DIF. Banbury et al. described the case of a 16-month-old infant with extremity swelling, palpable purpura, and previous Streptococcus pyogenes infection. The child developed ultrasound-proven intussusception, a rare HSP complication, and the biopsy confirmed LCV with neutrophilic and eosinophilic infiltration. Despite her symptoms, DIF with IgA was negative [13].

HSP in infants is commonly misdiagnosed as acute hemorrhagic edema of infancy (AHEI), a vasculitis of similar presentation with lower extremity purpura and edema [5]. While AHEI is more common in this age group, in our case, the associated systemic symptoms were more consistent with HSP [13]. In a study of 198 small-vessel vasculitis cases, of which 40 were clinically consistent with HSP, DIF was IgA-positive in 60% of cases, and C3 was the second most frequent deposit, often associated with renal symptoms. Other findings with positive IgA included lupus vasculitis, non-specific C3 deposition, and non-specific IgM deposition with subtle pathology changes in cases involving alternative deposits [11]. However, there is evidence to show IgA serum concentration playing a central role in the pathogenesis of HSP [7]. Positive complement C3 and IgA on DIF were found to be associated with renal involvement, further amplifying their role in disease progression. IgA abnormalities, particularly IgA1 glycosylation, also contribute to disease progression. Studies have identified children with HSP exhibiting a reduced sialic acid component in the IgA1 heavy chain hinge region, activation of alternative pathways of complement, and alterations of the galactose content of oligosaccharide-linked IgA1 glycosylation, which can affect clearance of IgA and lead to mesangial deposition [3,5,8]. 

The characteristic histologic finding of HSP is neutrophil infiltration in and around dermal vessels, scattered nuclear debris from degenerating neutrophils, and immune complex deposits indicating inflammatory cell recruitment [1,7,8,11]. The key mechanisms include galactose-deficient IgA1 immune complex formation, which promotes mesangial cell proliferation and pro-inflammatory cytokines such as tumor necrosis factor-alpha and interleukin-1 that drive neutrophil infiltration to sites of inflammation [8]. Immunofluorescence studies have revealed granular deposits of IgA alongside C3 and fibrin in lesser quantities within vessel walls, with findings more prominent in purpuric lesions but also detectable in normal skin. Lab tests for antinuclear antibody and IgM rheumatoid factor are frequently negative, but elevated cytokine abnormalities in urine samples have proven useful in identifying HSP [3]. 

In our case, despite negative DIF for IgA, the patient met the European League Against Rheumatism (EULAR) and American College of Rheumatology (ACR) criteria for HSP, which require clinical findings of lower extremity purpura in addition to one of the following symptoms: abdominal pain, histopathology-confirmed IgA deposition, arthralgias, age less than 20 years at onset, LCV confirmed by biopsy, or renal involvement [12,14]. Biopsies for other vasculitides have frequently been performed in patients without clinical suspicion for HSP. While histopathology can confirm LCV, identifying immune complexes within vessel walls aids in classifying vasculitis subtypes, including HSP [14]. However, DIF’s diagnostic value is limited, given that most vasculitides exhibit nonspecific DIF reactivity, with HSP being an exception [12]. Therefore, DIF results should be interpreted in conjunction with clinical findings, as its utility remains incompletely validated [14, 15].

Other small vessel vasculitides such as Wegener’s granulomatosis (WG), polyarteritis nodosa (PAN), and microscopic polyangiitis (MPA) can exhibit histologic parallels to HSP [16]. Distinguishing HSP from WG, PAN, and MPA is critical as these conditions can also share overlapping clinical features. DIF is a crucial laboratory technique and diagnostic tool that allows the visualization of antigen-bound antibodies and immune complexes in tissue and serves to differentiate WG and MPA from HSP [3,11,15]. For example, MPA typically lacks immune deposits on DIF, while IgG antineutrophil cytoplasmic antibody (ANCA) is commonly found in WG and MPA; however, this is not characteristic of HSP [3,17]. These findings highlight DIF’s complexity in differentiating between small vessel vasculitides. Given this variability, it is imperative to employ a multifactorial approach to diagnose HSP. 

Variability in DIF positivity criteria contributes to diagnostic challenges, as many studies note a lack of explicit definition. Generally, a positive DIF result is interpreted as a deposition of one or more immune reactants. Although evidence is conflicting, lesion age may affect DIF results; one study found specific immunoglobulin depositions by DIF in LCV biopsies to be dependent on lesion age [12]. Another review found no statistical difference between DIF results based on lesion age but did observe histopathology changes [15]. Studies with larger sample sizes have reported DIF sensitivity declining with increased lesion age. A cohort examining 198 suspected HSP cases found that biopsy timing significantly affected DIF results, noting that immune complex degradation and clearance can occur typically within 48-72 hours after the onset of skin lesions, thereby altering DIF results [11]. 

Studies evaluating DIF sensitivity in the diagnosis of vasculitis have shown mixed results [15]. A review of 182 vasculitis cases (92 IgA-positive) found that 27% of cases were falsely diagnosed on DIF alone, with erythema nodosum, urticaria, dermatitis, and purpura simplex among the misdiagnosed [14]. Moreover, several studies have observed IgA deposition in entities other than HSP, including LCV, eosinophilic granulomatosis with polyangiitis, urticarial vasculitis, coagulopathic vasculopathy, cryoglobulinemia, and livedoid vasculopathy [14,15]. Additional factors influencing the sensitivity of DIF include transport medium and lesion selection. A study of 65 patients found that lesional biopsies more often demonstrated vascular deposits on DIF than perilesional samples [14]. Another study assessing DIF discrepancies in 20 suspected HSP cases found IgA deposition in 75% of lesional biopsies vs. 67% of nonlesional biopsies [15]. Regarding biopsy location, immune complexes preferentially deposit in the lower extremities, making this a preferred biopsy site despite practical challenges. Moreover, the lower extremities frequently show nonspecific immune complex deposition due to hemodynamic factors [14]. Studies comparing biopsy preservation indicate that Michel’s medium yields higher DIF positivity rates (100%) compared to formalin (70%) [15]. While DIF can aid in vasculitis classification, its sensitivity is influenced by numerous factors, reinforcing the need for a multimodal diagnostic approach. 

Predicting systemic involvement in HSP remains a challenge. While IgA deposition has been found to correlate with renal disease in some studies, no consistent DIF pattern perfectly predicts systemic involvement. The association between IgA deposits and renal disease risk is well-documented [12,15], but studies differ in their assessment of DIF utility. One study found that gastrointestinal and renal involvement in younger generations were significantly more common in those with positive IgA deposition and confirmed HSP diagnosis [14]. Further investigation showed DIF positivity to be fairly similar across inflammatory phases, reporting that early stage, fully developed, and healing stage LCV exhibited the same sensitivity to DIF testing [15]. A longitudinal review found that DIF had a positive predictive value of 84% and a negative predictive value of 81% for HSP, and the cases in this review were eventually diagnosed with HSP due to clinical suspicion [12]. Sample size limitations in retrospective studies and variable HSP diagnostic criteria may affect the generalizability of these findings. Additionally, many studies were conducted in large, multispecialty tertiary centers, potentially limiting their applicability to broader populations. 

Initial evaluations of suspected HSP generally include urinalysis to detect proteinuria and hematuria, serum creatinine measurement, and blood pressure monitoring. Treatment strategies include symptom management with topical or systemic steroids, analgesics, diuretics, petrolatum to promote healing of necrotic lesions, and supportive care. Nonsteroidal anti-inflammatory drugs are encouraged but should be used with caution in patients with renal insufficiency and intestinal hemorrhage. In cases of renal involvement, corticosteroids may be ineffective in preventing nephritis but can reduce systemic symptom severity. Dapsone has also shown efficacy in reducing disease duration and IgA-neutrophil interactions [3]. Except for chronic nephritis, HSP is acute in nature, lasting anywhere from several days to several weeks. [3,4]. Although HSP can recur, relapses are usually more common in patients with elevated ESR at the time of diagnosis. While nephritis poses long-term risks, most studies indicate favorable renal outcomes and extrarenal symptoms resolving in three to eight weeks, even in cases with nephropathy [1,4]. HSP is typically self-limiting with an excellent prognosis, but renal involvement necessitates careful monitoring [3].

## Conclusions

DIF has a valuable role in diagnosing HSP by detecting vascular IgA deposits, though its sensitivity and specificity are not absolute. While skin biopsies from involved areas may enhance diagnostic accuracy, clinical correlation remains essential. DIF should always be interpreted alongside clinical and histopathological findings to ensure the best outcomes. Nephrological monitoring and early recognition are crucial to prevent complications. Further studies are needed to optimize treatment strategies and deepen our understanding of HSP’s pathogenesis, ultimately advancing patient care. A comprehensive diagnostic approach that integrates DIF with clinical presentation and histology can aid in timely intervention, ultimately improving patient outcomes.
